# OzTracs: Optical Osmolality Reporters Engineered from Mechanosensitive Ion Channels

**DOI:** 10.3390/biom12060787

**Published:** 2022-06-04

**Authors:** Thomas J. Kleist, I Winnie Lin, Sophia Xu, Grigory Maksaev, Mayuri Sadoine, Elizabeth S. Haswell, Wolf B. Frommer, Michael M. Wudick

**Affiliations:** 1 Institute for Molecular Physiology, Heinrich Heine University, 40225 Düsseldorf, Germany; kleistt@hhu.de (T.J.K.); mayuri.sadoine@hhu.de (M.S.); frommew@hhu.de (W.B.F.); 2 Department of Plant Biology, Carnegie Institution for Science, Stanford, CA 94305, USA; winnielinyi@gmail.com (I.W.L.); sophiax@wi.mit.edu (S.X.); 3 Department of Biology, Stanford University, Stanford, CA 94305, USA; 4 Department of Biology, Massachusetts Institute of Technology, Cambridge, MA 02142, USA; 5 NSF Center for Engineering Mechanobiology, Department of Biology, Washington University in St. Louis, St. Louis, MO 63130, USA; gmaksaev@wustl.edu (G.M.); ehaswell@wustl.edu (E.S.H.); 6 Institute of Transformative Bio-Molecules (WPI-ITbM), Nagoya University, Nagoya 464-8601, Japan

**Keywords:** sensor, mechanosensitive protein, osmolality, yeast

## Abstract

Interactions between physical forces and membrane proteins underpin many forms of environmental sensation and acclimation. Microbes survive osmotic stresses with the help of mechanically gated ion channels and osmolyte transporters. Plant mechanosensitive ion channels have been shown to function in defense signaling. Here, we engineered genetically encoded osmolality sensors (OzTracs) by fusing fluorescent protein spectral variants to the mechanosensitive ion channels MscL from *E. coli* or MSL10 from *A. thaliana*. When expressed in yeast cells, the OzTrac sensors reported osmolality changes as a proportional change in the emission ratio of the two fluorescent protein domains. Live-cell imaging revealed an accumulation of fluorescent sensors in internal aggregates, presumably derived from the endomembrane system. Thus, OzTrac sensors serve as osmolality-dependent reporters through an indirect mechanism, such as effects on molecular crowding or fluorophore solvation.

## 1. Introduction

Organisms experience diverse mechanical forces arising from their internal or external environments. Internally, cells and cellular components generate forces during cell division, elongation, and movement. Externally, both unicellular and multicellular organisms face constantly changing environments that frequently present mechanical and osmotic stresses. Soil-dwelling organisms experience extreme cycles of osmolality changes due to, for example, soil drying in the sun or rehydration during rainfall [1]. To be able to sense and respond to such changes, organisms evolved mechanosensors that monitor and respond to membrane tension changes. Mechanosensitive (MS) ion channels embedded in membranes are capable of detecting mechanical forces in membranes [2,3]. MS ion channels have been broadly categorized as either tethered (e.g., to the cytoskeleton or cell wall/extracellular matrix) or intrinsically sensitive to membrane tension. Channels gated directly by membrane tension play a critical role when, for example, unicellular organisms such as bacteria must maintain membrane integrity when confronted with osmotic stresses. During hypoosmotic stress, bacterial MS channels open directly in response to increased membrane tension and mediate ion efflux causing a passive extrusion of water from the cytosol, thereby averting cell swelling [4,5]. These “osmotic safety valves” mediate solute efflux during hypoosmotic shock to protect cells from lysis caused by excess membrane tension [6,7]. Bacterial MS channels, especially MscL and MscS (mechanosensitive channel of large and small conductance, respectively), have been characterized extensively. Structurally, *Ec*MscL (MscL from *E. coli*) and homologs form nonselective ion channels as pentamers with two transmembrane (TM) helices [8,9]. *Ec*MscS assembles as a homo-heptamer, with each subunit containing a short N-terminal periplasmic domain, three TM helices per subunit, and a C-terminal cytoplasmic region [10,11]. Both the MscS and MscL channel families are typified by large pores with diameters of roughly 13 and 30 Å, respectively [12,13], whose osmolarity-dependent gating and subsequent release of ions and metabolites prevent cell rupture.

The MscS family is much larger than the MscL family and includes homologs from eukaryotes, unlike the MscL family. The MscS family is more variable in size and sequence across different species than the MscL family [14]. Much of the diversity in the MscS family stems from variation in the number of TM helices per subunit, ranging from three to eleven. A family of ten MscS-like channels (MSL) in Arabidopsis (*Arabidopsis thaliana*) exhibits discernible structural homology to the pore and adjacent β-domain of *Ec*MscS but also contains diverse domains and topologies outside the pore-lining domain and adjacent regions [15]. Some of the biological functions of eukaryotic MscS homologs have been characterized. For example, Msy1 and Msy2 from *Schizosaccharomyces pombe* localize to the endoplasmic reticulum (ER) membrane, where they influence cytosolic calcium (Ca^2+^) elevation in response to hypoosmotic shock [14]. In Arabidopsis, MscS-Like 1 (*At*MSL1), whose structure has been recently solved by cryo-electron microscopy [16], localizes to the inner mitochondrial membrane where it is suggested to contribute to the dissipation of the mitochondrial membrane potential under abiotic stress [17]. *At*MSL2 and *At*MSL3, as well as MSC1 from the alga *Chlamydomonas reinhardtii* [18], localize to the plastid envelope, where they may provide stretch-activated channel activity gated in response to osmotic imbalance between stroma and cytoplasm [5,15,19]. The plasma membrane (PM)-localized *At*MSL8 is a pollen-specific membrane tension–gated ion channel essential for male fertility and required for pollen survival under hypoosmotic stress associated with rehydration [20]. Arabidopsis MSL9 and MSL10 localize to the PM, and their gating properties have been studied in root protoplasts [5] and *Xenopus laevis* oocytes [21]. Nonetheless, *msl9;msl10* double mutants and *msl4;msl5;msl6;msl9;msl10* quintuple mutant plants, which each lacked measurable stretch-activated channel activity, responded normally to various mechanical and osmotic stresses and showed no obvious phenotypic defects [5]. Recently, *At*MSL10 was shown to function in cell swelling responses [22], transduction of oscillatory mechanical signals [23], and long-distance wound signaling [24]. *At*MSL10 has been posited to act as a hydraulic sensor in plant vascular bundles, but the precise mechanism for *At*MSL10 activation during wound signaling is not fully understood.

Genetically encoded fluorescent biosensors have served as transformative tools for biologists in recent decades. Placement of sensory domains or full-length proteins between fluorescent protein domains spectrally compatible for Förster resonance energy transfer (FRET) has proven a facile and effective strategy for engineering genetically encoded biosensor prototypes [25,26]. FRET efficiency is strongly dependent on the distance and relative orientation of the dipole moments of the chromophores. Therefore, conformational changes of a sensory domain commonly cause changes in the FRET efficiency. The goal of this study was to construct a genetically encoded integral membrane fluorescent reporter for osmotic potential and/or mechanical force. We chose a variety of mechanosensitive membrane proteins and used a sensor plasmid library consisting of a variety of previously tested FRET pairs [27] to generate candidate sensors for expression in yeast (*Saccharomyces cerevisiae*). Unlike many soluble proteins, sensors constructed from integral membrane proteins are challenging to purify and work with in vitro. Thus, to screen for candidate membrane mechanosensors, live yeast cells expressing fluorescent chimeras were treated with solutions of varying osmotic potential and were monitored by fluorescence spectroscopy. Identified osmolality tracking or ‘OzTrac’ sensors based on *Ec*MscL or *At*MSL10 reported dose-dependent osmolyte treatments and were further characterized by trapped-cell microfluidics and confocal fluorescence microscopy, revealing sensor reversibility upon osmolyte removal. The *At*MSL10-derived OzTrac sensor retained mechanosensitive ion channel activity when expressed in frog (*Xenopus laevis*) oocytes, demonstrating the functionality of the OzTrac chimera and its presence in the PM. In contrast, the OzTracs aggregated internally when expressed in yeast, where they nonetheless function as sensors for osmotic potential. We hypothesize that the sensor mechanism is unrelated to membrane tension but rather is caused by molecular crowding and/or changes in fluorophore solvation.

## 2. Materials and Methods

### 2.1. Plasmid Constructs

The full-length open reading frames (ORFs) of MSL10 (At5g12080), AHK1 (At2g17820), and OSCA1.1 (At4g04340) from *A. thaliana* and MscL (JW3252) from *E. coli* were cloned into the TOPO GATEWAY entry vector. The yeast expression vectors were created by GATEWAY LR reactions between pTOPO plasmids and the pDRFLIP-GW yeast expression vector series [27], carrying fluorescent proteins of a FRET pair flanking the Gateway cassette (Table S1) following the manufacturer’s instructions. For assays in *Xenopus laevis* oocytes, cDNAs were cloned into the oocyte expression vector pOO2-GW [21].

### 2.2. Expression of Sensors in Protease-Deficient Yeast

The protease-deficient yeast strain BJ5465 (*MAT*a *ura3–52 trp1 leu2*Δ*1 his3*Δ*200 pep4:HIS3 prb1*Δ*1.6R can1*) was obtained from the Yeast Genetic Stock Center (University of California, Berkeley, CA, USA). Transformation of yeast cells was performed using a lithium acetate method [28]. Transformants were selected on synthetic media containing yeast nitrogen base (YNB, Difco) supplemented with 2% glucose and DropOut supplements lacking uracil (Clontech, Mountain View, CA, USA). Single colonies were used to inoculate 5 mL of liquid YNB media supplemented with 2% glucose and DropOut supplements lacking uracil. Cells were grown with agitation (230 rpm) at 30 °C overnight until an OD_600nm_ of 0.2–0.3 was reached. Liquid cultures were sub-cultured by dilution to an OD_600nm_ of 0.1 in the same medium and grown at 30 °C with agitation until the cultures reached OD_600nm_ ∼ 0.4. Cells were harvested by centrifugation for further analysis.

### 2.3. Fluorimetry

Experiments were performed similarly to previous publications [27,29,30]. Briefly, fresh yeast cultures (OD_600nm_ ∼ 0.4) were washed three times in 50 mM 2-(*N*-morpholino)-ethanesulfonic acid (MES) pH 5.5 and resuspended in 50 mM MES pH 5.5. Fluorescence was measured with a fluorescence plate reader (M1000; TECAN, Grödig/Salzburg, Austria) in bottom reading mode using 7.5 nm bandwidths for both excitation and emission [31,32]. To quantify the fluorescence responses of the sensors to different osmolytes, 100 µL aqueous solutions containing 50 mM MES pH 5.5 were added to 100 µL of cell suspension in 96-well flat-bottom plates (#655101; Greiner, Monroe, NC, USA). Osmolyte titration curves were analyzed using the GraphPad Prism software (version 9). Data represent means and standard errors of the mean (SEM) of three technical replicates. Dose-response curves were fitted to a non-linear regression with a Hill slope and a constant: Y = B_max_ (X^h^)/(EC_50_^h^ + X^h^) + a, with B_max_ being the maximum specific response, EC_50_ the half-maximal effective concentration, and h the Hill slope, which describes the cooperativity of the response, and a constant (a) added to the equation.

### 2.4. Electrophysiology

*Xenopus laevis* oocytes were injected with complementary RNAs (cRNAs) encoding OzTrac-MSL10-34 that were transcribed from linearized pOO2-GW-OzTrac-MSL10-34 plasmid. Each oocyte was injected with approximately 50 ng of cRNA. Excised inside-out patches were bathed symmetrically in 60 mM magnesium chloride (MgCl_2_), 10 mM 4-(2-hydroxyethyl)-1-piperazineethanesulfonic acid (HEPES), pH 7.4, and were subjected to pressure ramping from 0–140 mm (maximum) of mercury (mmHg). The electrodes pulled from glass capillaries had a final resistance of 3–4 MOhm. The experiments were typically performed within 3–5 days after oocytes injection [33].

### 2.5. Fluorescence Microscopy

Quantitative imaging was performed on a spinning disk confocal microscope. Yeast cells were trapped as a single cell layer in a microfluidic perfusion system (Y04C plate, Onyx, CellASIC, Hayward, CA, USA) and perfused with media containing 50 mM MES pH 5.5 with or without specified osmolytes. Microscopy data were acquired using an Olympus IXplore SpinSR confocal microscope equipped with UPLSAPO 100 × 1.35 NA silicone immersion oil objective (Olympus), 50 µm spinning disk, and dual Photometrics Prime BSI sCMOS cameras. Acquisitions were performed using 2 × 2 pixel binning. A 75 mW 445 nm OBIS LX laser was used as an excitation source for DxDm and DxAm acquisitions, and a 40 mW coherent OBIS LX 514 nm laser was used to excite the AxAm channel. A 445/514/640 nm dichroic and 514 nm long pass beam splitter were used. For DxDm acquisition, a 482/35 nm emission filter was used, and a 534/23 nm emission filter was used for DxAm and AxAm acquisitions. A Nano-ZL300-OSSU fast piezo stage (Mad City Labs, Madison, WI, USA) was used for z-stack acquisition, and an IX3 ZDC2 (Olympus) was used for z-drift compensation. Average z-stack projections were performed in ImageJ [34] prior to data analysis and figure preparation. Ratiometric images were prepared for display using a 16-color lookup table and binary mask made from the AxAm channel in ImageJ.

### 2.6. Yeast Growth Assays

The *S. cerevisiae* strains used for hypoosmotic stress assays were BY4743 (*MAT*a*/α his3*Δ*1*/*his3Δ1 leu2*Δ*0*/*leu2*Δ*0 LYS2*/*lys2*Δ*0 met15*Δ*0*/*MET15 ura3*Δ*0*/*ura3*Δ*0*), and the HomoDip knock out *fps1Δ* mutant in the same genetic background (YLL043W, clone ID: 31531, GE Dharmacon). Yeast cells were transformed with OzTrac-MscL36 or OzTrac-MSL10 using a lithium acetate-based method [28], and transformants were selected on synthetic medium lacking uracil (Clontech, Mountain View, CA, USA) at 30 °C. For hyperosmotic culture conditions, yeast cells were grown in liquid medium supplemented with 1 M sorbitol. For hypoosmotic shock treatments, yeast cells were transferred to liquid medium without sorbitol [35].

### 2.7. Structural Representations

Modeling of the structures of sensors was performed using UCSF Chimera software (UCSF, San Francisco, CA, USA) [34]. Protein structures were obtained from RCSB Protein Data Base (PDB): CFP (2WSN), YFP (1YFP), *Mycobacterium tuberculosis* MscL (*Mt*MscL, 2OAR), and the three-dimensional structure of *At*MSL10 was generated in a previous study [24].

## 3. Results and Discussion

### 3.1. Construction and Characterization of Membrane Mechanosensors

In organisms equipped with cell walls such as bacteria, fungi, and plants, the plasma membrane (PM) is typically under tension caused by turgor pressure.

**Figure 1:**
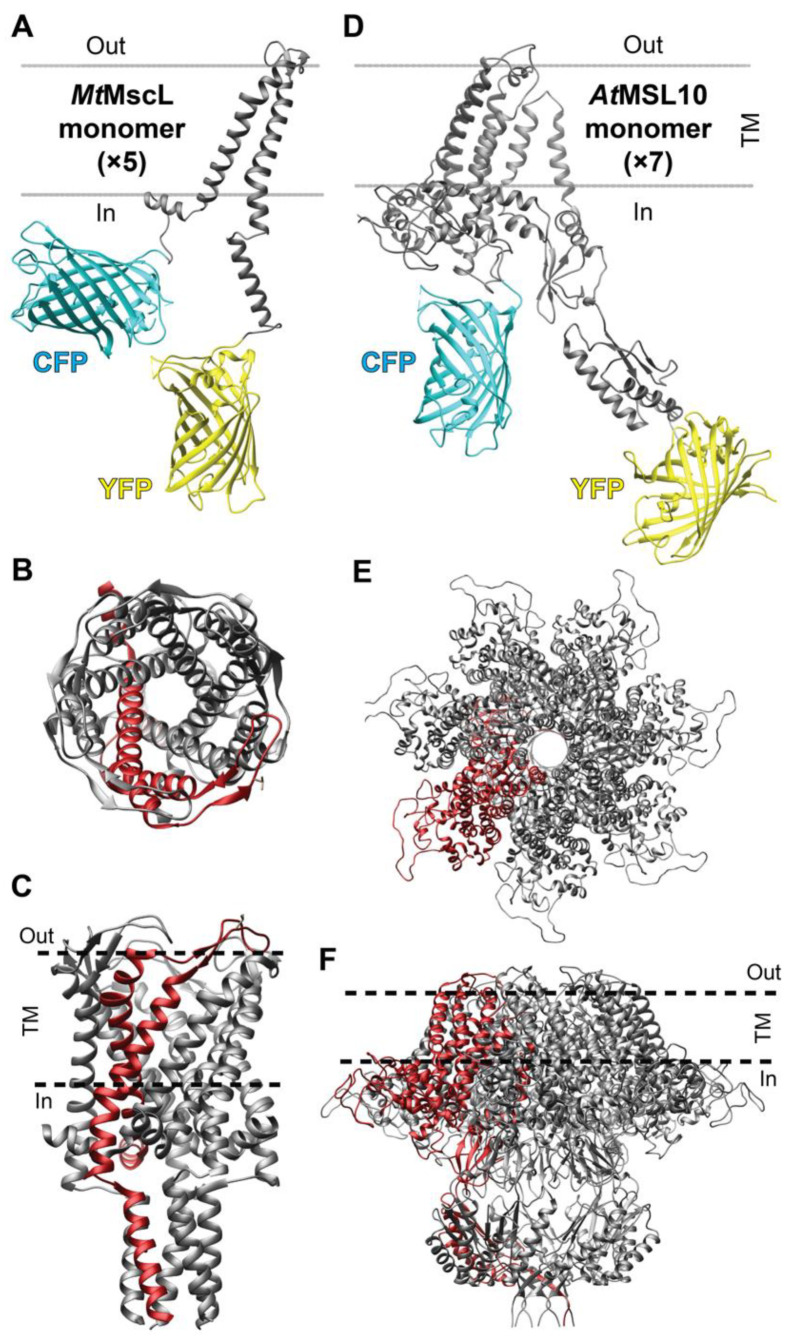
Monomeric structural representations of OzTrac sensors. Sensors were engineered by cloning the coding sequences of *Mt*MscL (**A**–**C**) and *At*MSL10 (**D**–**F**) between an N-terminal cyan fluorescent protein (CFP) and C-terminal yellow fluorescent protein (YFP). *Mt*MscL and *At*MSL10 are predicted to assemble as pentamers (top-view in (**B**), side-view in (**C**)) and heptamers (top-view in (**D**), side-view in (**F**)), respectively. Visualization of the structures was performed using UCSF Chimera software, and the three-dimensional structure of *At*MSL10 was generated in a previous study. TM: transmembrane domain.

Mechanical forces in the PM can therefore be affected by changing extracellular osmotic potential. Specifically, membrane tension can be reduced by treatment with hyperosmotic extracellular solutions or increased by treatment with hypoosmotic extracellular solutions. With the aim of constructing a membrane tension sensor, we fused FRET-compatible variants of cyan and yellow fluorescent proteins with integral membrane proteins reported to function as mechanosensitive, PM-localized proteins (Figure 1, Tables S1 and S2, Supplementary Materials).

Candidate sensory domains included the mechanosensitive ion channels *Ec*MscL and *At*MSL10. Chimeric sensors were expressed in *S. cerevisiae* cells, and fluorescence from live cells was monitored under hyperosmotic stress or isosmotic control conditions. The OzTrac sensor responses were estimated as a change in the ratio of putative FRET acceptor emission (Am) to putative FRET donor emission (Dm) under FRET donor excitation (Dx): DxAm/DxDm. As the largest ratio changes were observed for the *Ec*MscL- and *At*MSL10-based sensors, further characterization focused on those variants.

### 3.2. Characterization of Sensors Constructed with EcMscL

**Figure 2:**
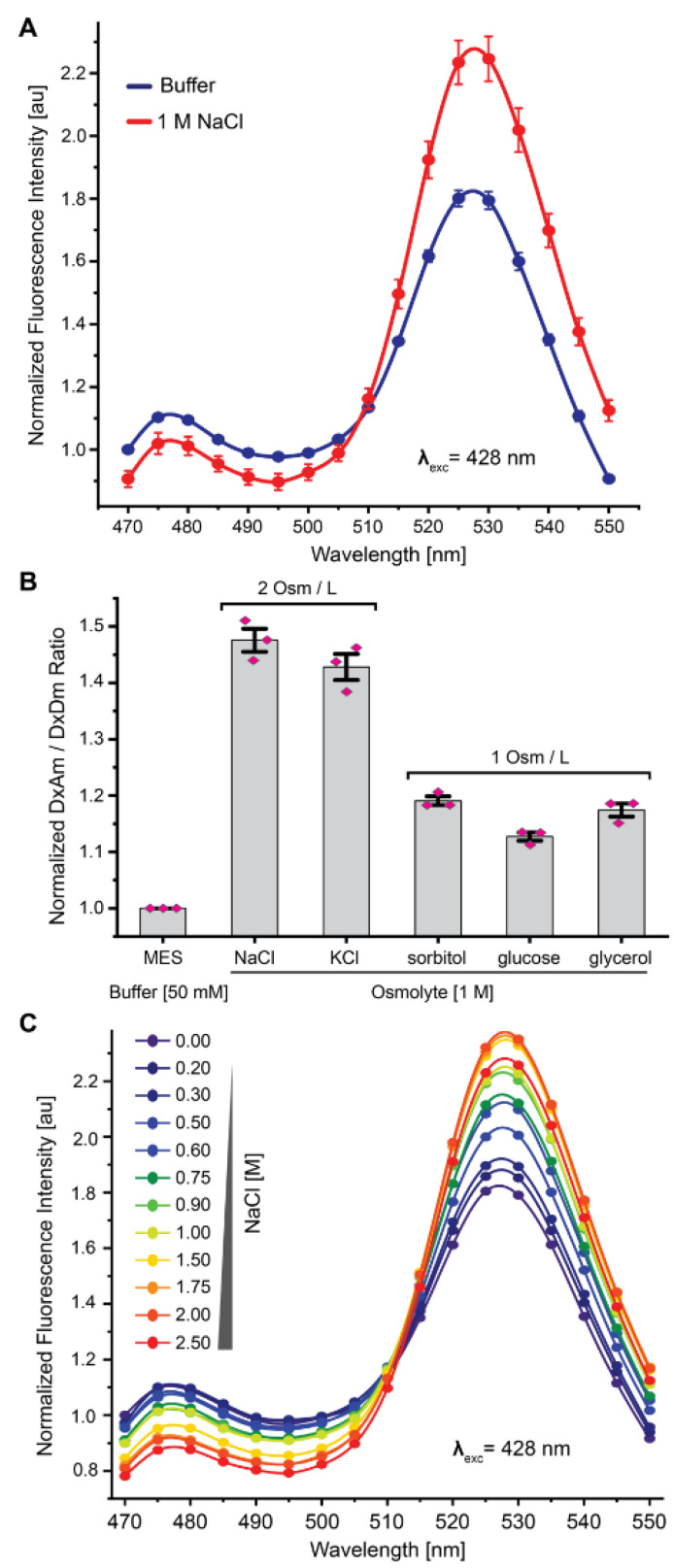
Characterization of the OzTrac-MscL-36 sensor. (**A**) Emission spectra of yeast cells expressing OzTrac-MscL-36 exposed to buffer with or without NaCl. X-axis: emission wavelength in nanometers (nm). Y-axis: fluorescence intensity normalized to 470 nm emission under control conditions. Excitation wavelengths (λ_exc_) are shown. (**B**) Various osmolytes, such as NaCl, KCl, sorbitol, glucose, or glycerol elicit ratiometric changes approximately proportional to solution osmolality (indicated as Osm/L). (**C**) Concentration-dependent effects of osmolyte (NaCl) treatment. Axes and inset same as in Figure 1A. All error bars: SEM.

The OzTrac candidates based on *Ec*MscL showed the largest DxAm/DxDm ratio changes with 3–45% greater DxAm/DxDm ratios when treated with 1 M sodium chloride (NaCl, 2 Osm/L) (Table S1). Direct effects on the acceptor fluorophore (i.e., acceptor emission under direct acceptor excitation (AxAm)) were not substantial (Figure 2A, Figure S1A and Table S1, Supplementary Materials). Within the *Ec*MscL subgroup, the Aphrodite–Cerulean fluorophore combination (OzTrac-MscL-36) yielded the largest response with a +50% ratio change (DxAm/DxDm) when cells were treated with 1 M NaCl and was selected for further investigation in yeast.

To exclude that the observed ratio change could be caused by ion-specific effects on either the fluorophores or cellular host, other osmolytes were tested at 1 M concentration, including potassium chloride (KCl), sorbitol, glycerol, and glucose (Figure 2B). The addition of 1 M (2 Osm/L) KCl triggered a ratio change similar to that found for equimolar NaCl addition (~45%). Addition of 1 M (1 Osm/L) sorbitol, glucose, or glycerol elicited lower ratio changes (+~15–20%). To estimate the dynamic response range of OzTrac-MscL-36, yeast cells expressing OzTrac-MscL-36 were treated with NaCl concentrations ranging from 0–2.5 M, and concentration-dependent spectral changes were observed for treatments with up to 1.75 M NaCl before reaching saturation (Figure 2C and Figure S1B, Supplementary Materials), suggesting that OzTrac-MscL-36 can report changes in extracellular osmotic potential over a wide range.

### 3.3. Characterization of Sensors Constructed with AtMSL10

Plants lack discernible MscL homologs but are equipped with homologs of MscS. Previous work characterized Arabidopsis MSL10 by patch clamp in root protoplasts and oocytes and showed that it localized to the PM [5,21]. In excised membrane patches, *At*MSL10 can be opened by application of positive or negative pipette pressure; channel closure occurs upon release of pressure. With the aim of creating a membrane tension sensor suitable for deployment in plants, we engineered fluorescent sensors based on *AtMSL10*, which was cloned into destination vectors containing 13 different fluorophore pairs [27] (Table S1, Supplementary Materials), and named the candidates with the two largest response ratio changes (Aphrodite.t9-t7.mTFP.t9 and Aphrodite.t9-mTFP.t9) as OzTrac-MSL10–34 and OzTrac-MSL10-35, respectively.

**Figure 3:**
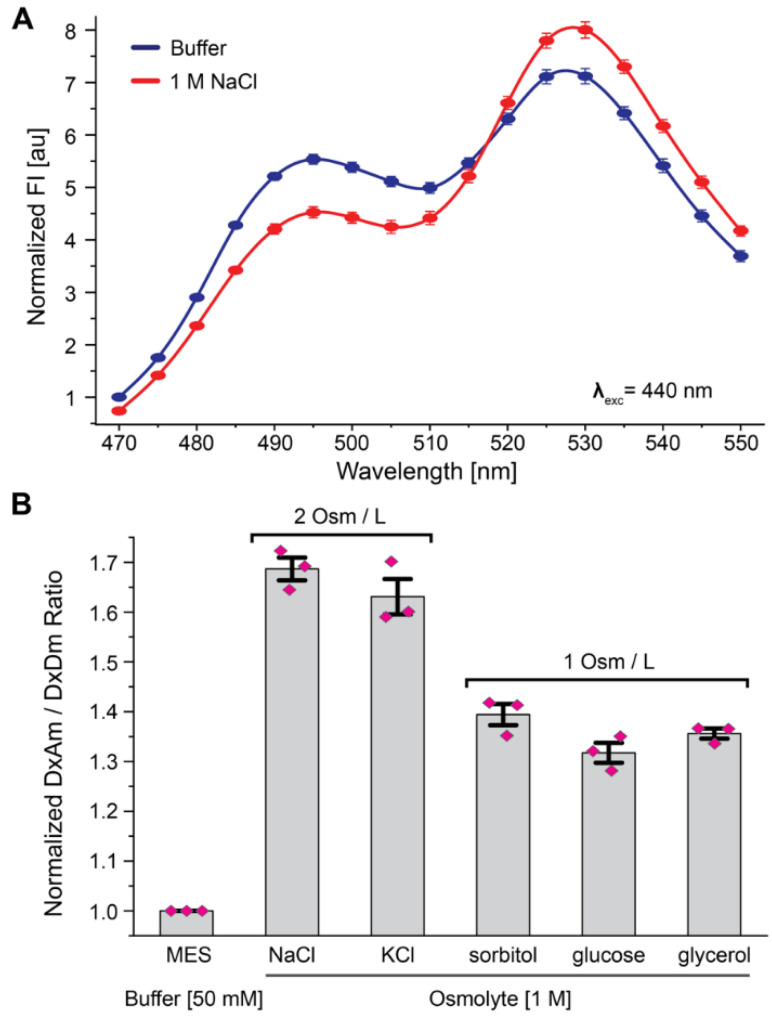
Characterization of the OzTrac-MSL10-34 sensor. (**A**) Emission spectra of yeast cells expressing OzTrac-MSL10-34 exposed to buffer with or without NaCl. (**B**) Response to osmolytes: NaCl, KCl, sorbitol, glucose, or glycerol (DxAm/DxDm) elicit ratiometric changes approximately proportional to solution osmolality (indicated as Osm/L). Axes and formatting as in Figure 2.

Each sensor exhibited an ~70% increase in ratio change when treated with 1 M NaCl (Figure 3A, Figure S1C and Table S1, Supplementary Materials). Due to the similar responses and our interest in deployment in plants, further sensor characterization focused only on OzTrac-MSL10-34.

Similar to OzTrac-MscL-36, diverse osmolytes were able to elicit OzTrac-MSL10-34 DxAm/DxDm ratio changes (Figure 3B). The addition of 2 Osm/L KCl showed a similar ratio of DxAm/DxDm changes as NaCl (60–70% increase), whereas the addition of 1 Osm/L sorbitol, glucose, and glycerol yielded smaller changes in the ratio (30–40% increase) compared to NaCl and KCl, respectively.

**Figure 4:**
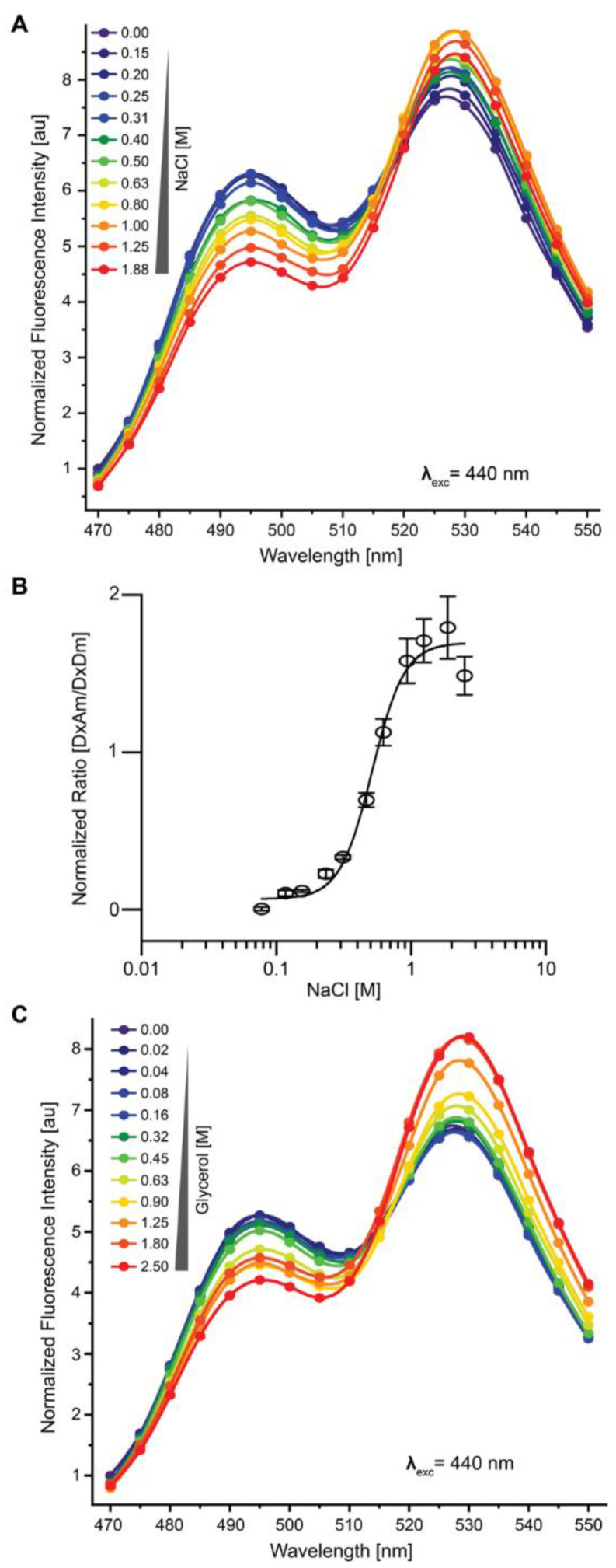
Osmolality-dependent ratio changes of OzTrac-MSL10-34 in yeast. (**A**) Response of the sensor to different NaCl concentrations. (**B**) Dose-response curve of OzTrac-MSL10-34 fitted to a non-linear regression with a Hill slope with a half-maximal effective concentration EC_50_ of 573 ± 85 mM. (**C**) Response to the sensor to different glycerol concentrations. Axes as in Figure 2.

Treatment of cells containing OzTrac-MSL10-34 with a concentration gradient of NaCl ranging from 0–1.88 M revealed concentration-dependent spectral differences (Figure 4A and Figure S1D, Supplementary Materials). OzTrac-MSL10-34 effectively reported NaCl concentrations in the range of 200–800 mM with a half-maximal effective concentration (EC_50_) of 573 ± 85 mM (Figure 4B). Concentrations of NaCl > 0.8 M were associated with reduced fluorescence intensity from the acceptor fluorophore when exciting either the donor or the acceptor. Treatment with a gradient of glycerol concentrations ranging from 0–2.5 M similarly revealed concentration-dependent spectral effects (Figure 4C and Figure S1E, Supplementary Materials) with a working range from 0.32–1.8 M. *In planta*, *At*MSL10 functions as a stretch-activated ion channel that preferentially conducts anions [21]. To test whether stretch-activated channel activity is retained in the *At*MSL10-based OzTrac sensor, we characterized OzTrac-MSL10-34 by electrophysiology and yeast suppressor analysis.

### 3.4. Electrophysiological Analysis of OzTrac-MSL10-34

**Figure 5:**
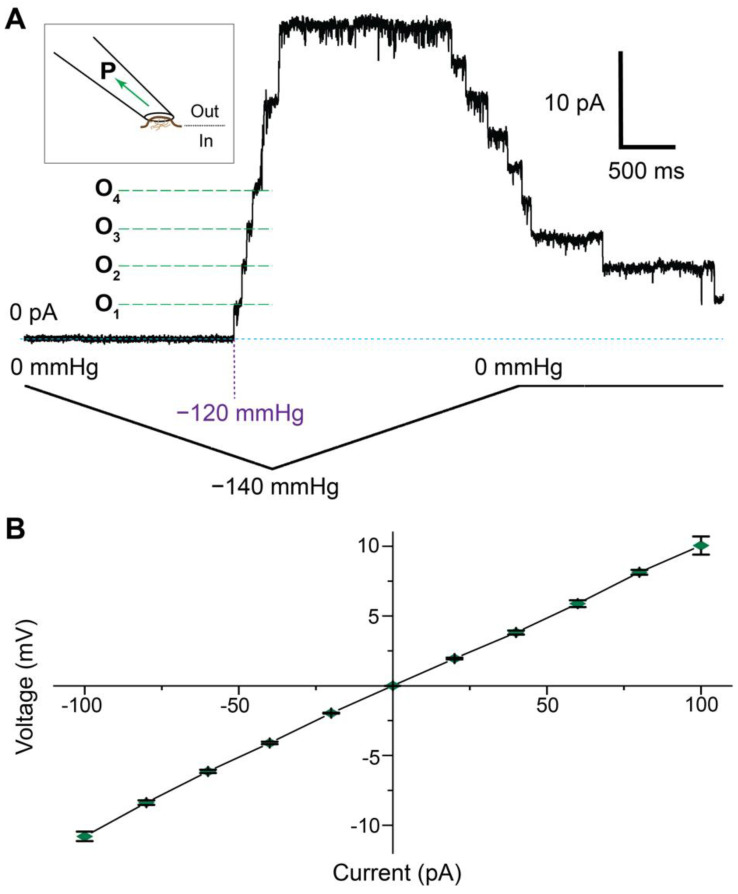
Ion channel activity of OzTrac-MSL10-34 expressed in *Xenopus laevis* oocytes. (**A**) Pressure ramp of an inside-out patch excised from oocytes. Schematic in upper left depicts excised patch of membrane; outer leaflet of the plasma membrane faces the interior of the pipette. Negative pressure (P) or suction was applied during patch clamp recordings. Example channel opening events (O_n_) are annotated and marked by green dotted lines, and pressure is given in millimeters of mercury (mmHg). Membrane potential = −40 mV. Note channel hysteresis, as previously described for *At*MSL10. (**B**) Current-voltage plot of currents observed in stretch-activated membrane patches. Unitary (single-channel) conductances were similar to published data for unmodified AtMSL10. Error bars: SEM (*n* = 2–17 excised patches).

To test whether the OzTrac-MSL10-34 sensors are fully functional and forms stretch-activated ion channels, the chimera was expressed in oocytes and analyzed by patch clamping. Excised inside-out patches showed ion channel activity in response to pressure ramping (Figure 5). Observed unitary conductances (~102 picosiemens [pS] at negative membrane potentials, ~80 pS at positive membrane potentials) were nearly identical to the previously reported values for *At*MSL10 [21], and gating pressure asymmetry (hysteresis), typical for wild-type *At*MSL10, was also observed. Together, the data indicate that OzTrac-MSL10-34 forms a functional ion channel and that the fluorescent tags do not substantially alter *At*MSL10’s channel properties or gating cycle.

### 3.5. Suppression of Yeast fps1Δ Mutant Phenotype by Expression of OzTracs

To test whether OzTrac-MscL-36 and/or OzTrac-MSL10-34 can function as ‘safety valves’ during hypoosmotic shock, as may be expected for functional mechanosensitive ion channels, the sensors were expressed in the yeast *fps1Δ* deletion mutant. The aquaglyceroporin Fps1 is required for survival in hypoosmotic shock conditions [36]. Cells expressing the sensors were grown in liquid medium containing 1 M sorbitol, and serial dilutions of yeast cultures were inoculated onto solid medium with or without sorbitol. While the *fps1Δ* mutant grew on isosmotic medium containing 1 M sorbitol, little growth was observed on hypoosmotic media lacking sorbitol (Figure S2, Supplementary Materials). Expression of either OzTrac-MscL-36 or OzTrac-MSL10-34 suppressed the *fps1Δ* phenotype by partially rescuing growth on media lacking sorbitol, consistent with the interpretation that OzTracs exhibit channel activity in yeast (Figure S2, Supplementary Materials). It is unknown whether OzTrac-MSL10-34 or OzTrac-MscL derivatives transport glycerol, such as FPS1, or whether transport of other osmolytes may be responsible for the observed phenotypic suppression.

### 3.6. OzTrac-MSL10-34 Likely Reports Molecular Crowding or Solvation Status

To assess the subcellular localization and reversibility of sensor response, we turned to spinning disk confocal microscopy of trapped yeast cells.

**Figure 6:**
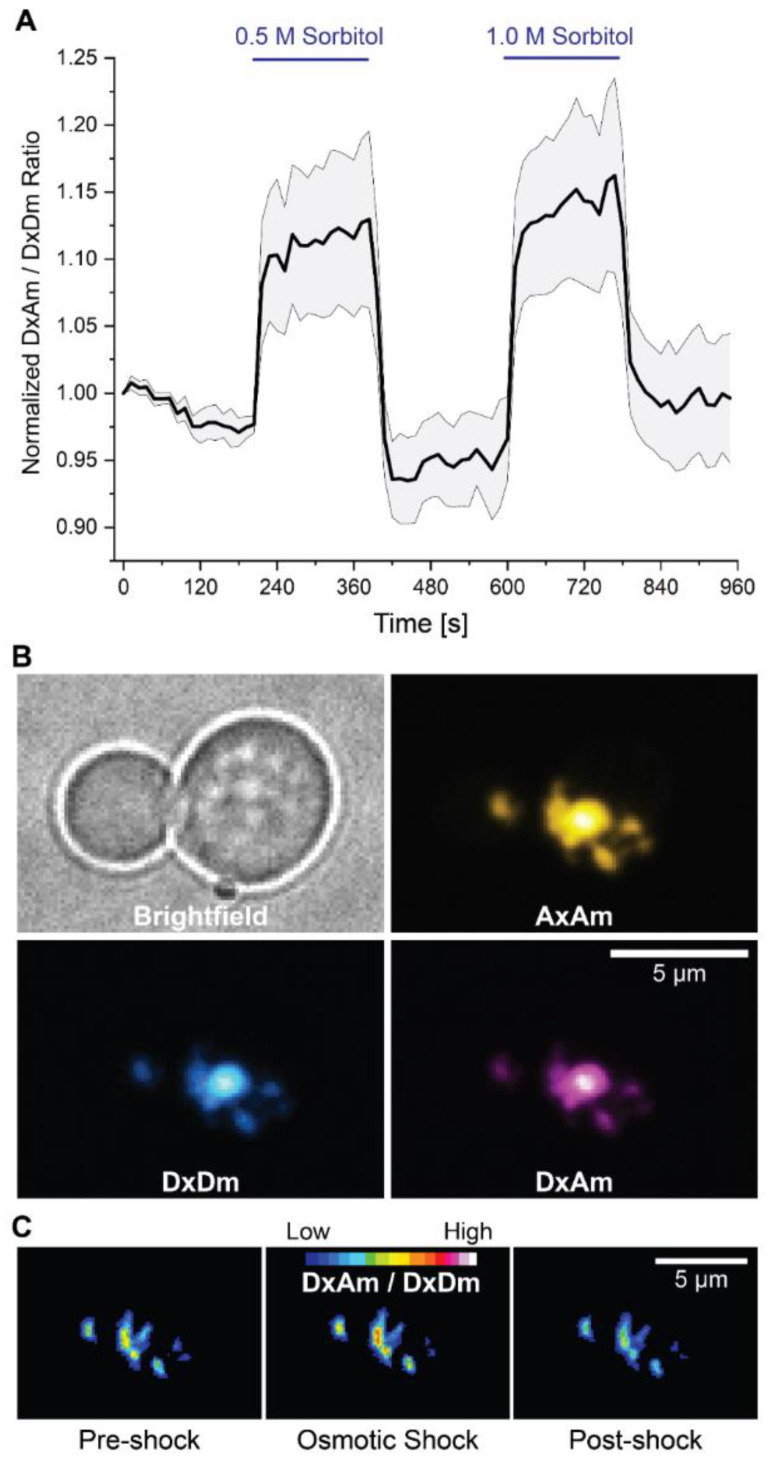
Quantitative fluorescence microscopy of yeast cells expressing OzTrac-MSL10-34. (**A**) Quantification of DxAm/DxDm ratio in trapped yeast cells exposed to square pulses of 0.5 or 1.0 M sorbitol (blue bars). Black line: mean (*n* = 5 different cells). Shaded region: SEM. (**B**) Example images of brightfield, AxAm, DxDm, and DxAm channels. Fluorescence images shown in “Hot” pseudocolor lookup tables (ImageJ). (**C**) Representative example of ratiometric (DxAm/DxDm) response from aggregates. A 16-color lookup table and a binary mask from AxAm channel were applied (see Materials and methods for details). Comparable results were obtained from 4 independent experiments.

Yeast expressing OzTrac-MSL10-34 were pressure-trapped in microfluidic devices and perfused with solutions of varying osmotic potential. Increased DxAm/DxDm ratios were elicited by treatment with 0.5 or 1.0 M sorbitol and could be reversed by osmolyte removal (Figure 6A). Strikingly, fluorescence was observed almost exclusively in internal structures that appeared as dense aggregates (Figure 6B).

We surmise that sensor-containing aggregates arise from the endomembrane system and may be a consequence of overexpression of membrane proteins, as previously reported [37]. Ratiometric imaging confirmed that changes in the DxAm/DxDm ratio occurred in internal puncta (Figure 6C). Although we cannot exclude the possibility that a fraction of the sensor was present in the PM, our interpretation is that the OzTrac response is likely unassociated with events at the PM or membrane tension. Rather, we hypothesize that molecular crowding or differential hydration induced by hyperosmotic stress (as a consequence of changes in extracellular osmolality) may cause the observed response of intracellular OzTracs. Interestingly, a cytosolic cage domain in bacterial MscS has been implicated as a sensor for macromolecular crowding [38].

## 4. Conclusions

In this study, we report construction of OzTracs, fluorescent sensors derived from mechanosensitive ion channels that report osmolality. OzTracs do not appear to target the cell membrane in yeast but rather aggregate internally. Localization to internal aggregates was unexpected given the suppression of the hypoosmotic shock-induced growth inhibition in the yeast *fps1Δ* strain. This apparent contradiction might be explained by the presence of a small population of sensors at the cell membrane below our fluorescence detection threshold. The mode of function for OzTracs is unclear; however, we posit that the sensors undergo FRET changes caused by molecular crowding and/or changes in protein hydration status. Irrespective of the mechanism, OzTrac-MSL10-34 and OzTrac-MscL-36 may be useful tools for tracking external osmolality in yeast. The findings reported here may also serve as a cautionary tale and help guide future strategies to pursue construction of fluorescent reporters for osmotic potential and/or mechanical force at the PM. Future work should include efforts to improve trafficking to the PM, for example, by nested insertion of one or more fluorescent protein domains within the channel moiety. This approach has been successfully employed for development of sugar sensor-transporter chimeras that target the yeast PM [39]. Given that we observed stretch-activated channel activity of OzTrac-MSL10-34 at the PM of *Xenopus leavis* oocytes, the sensor may be suitable for use in animal and plant cell membranes.

## Data Availability

Data supporting reported results can be found at: https://osf.io/sj8nf/?view_only=eb6c5ec11ea84ea7b9123f5b88f6c5f0.

## Supplementary Materials

The following supporting information can be downloaded at: https://www.mdpi.com/article/10.3390/biom12060787/s1, Figure S1: Acceptor fluorophore emission spectra under direct excitation (AxAm), Figure S2: Partial suppression of the growth phenotype of the yeast plasma membrane aquaglyceroporin mutant *fps1*Δ. Table S1: Fluorophore pairs in pDRFLIP destination vectors and observed DxAm/DxDm ratios when treated with 1 M NaCl. Table S2: Candidate proteins for a FRET membrane tension sensor [40,41,42].
